# Allergic bronchopulmonary mycosis characterized by chest pain and high‐attenuation mucus on computed tomography

**DOI:** 10.1002/ccr3.6030

**Published:** 2022-07-22

**Authors:** Risa Hirata, Masaki Tago, Seijiro Makio, Toru Oishi, Yoshimasa Oda

**Affiliations:** ^1^ Department of General Medicine Saga University Hospital Saga Japan; ^2^ Department of General Medicine Yuai‐Kai Foundation and Oda Hospital Saga Japan

**Keywords:** allergic bronchopulmonary aspergillosis, allergic bronchopulmonary mycosis, chest pain, high‐attenuation mucus

## Abstract

A 45‐year‐old woman developed chest pain on the previous day. High‐attenuation mucus in the bronchus was found on the CT examination on admission, which led to a diagnosis of allergic bronchopulmonary mycosis. CT should be checked carefully for high‐attenuation mucus because this finding is highly specific for allergic bronchopulmonary mycosis.

## Abstract 2

High‐attenuation mucus on CT examination leads to a diagnosis of allergic bronchopulmonary mycosis. Therefore, CT should be checked carefully for high‐attenuation mucus because this finding is highly specific for allergic bronchopulmonary mycosis.

## 1 CASE

**FIGURE 1:**
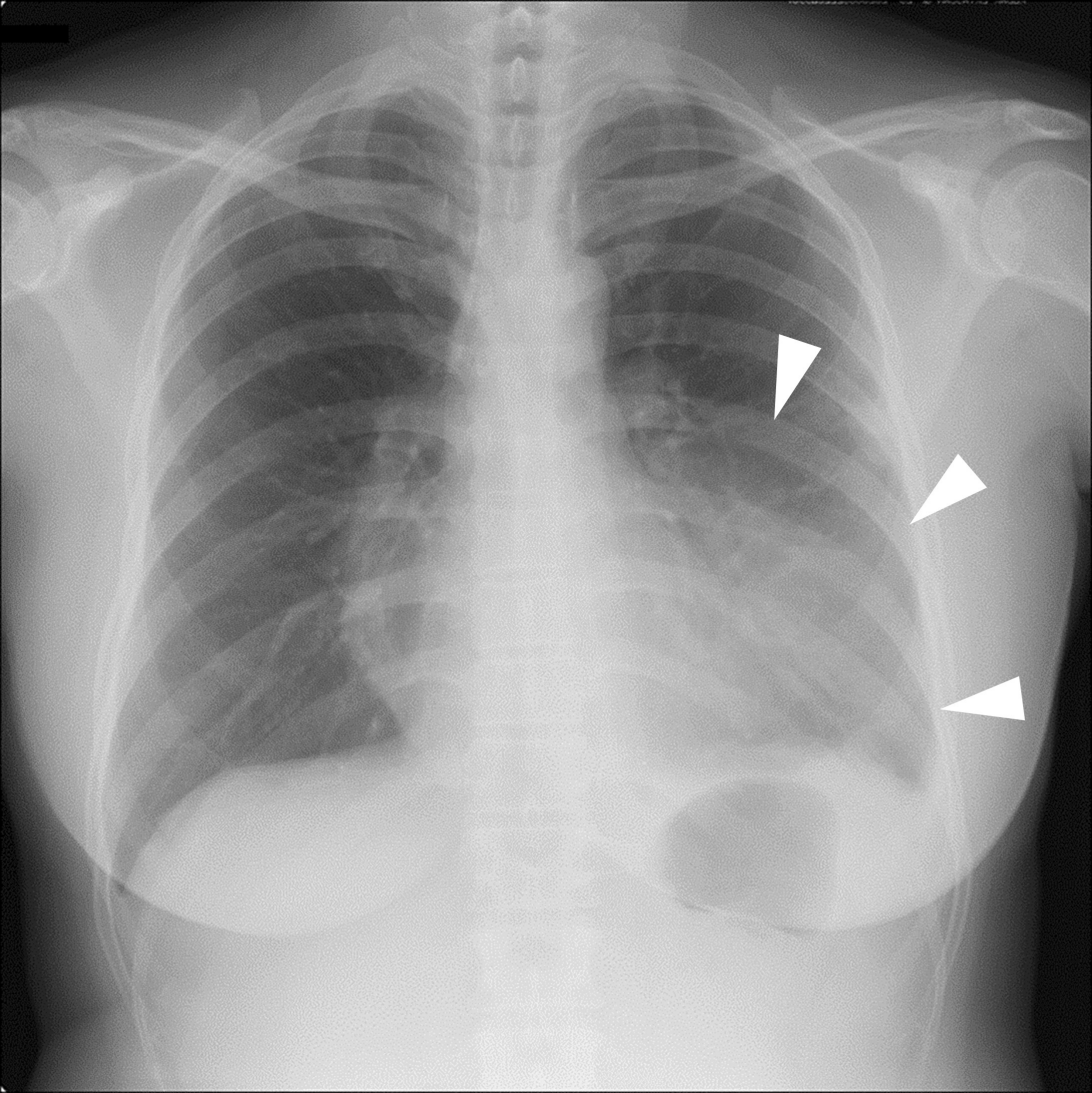
Chest radiograph obtained on admission. Infiltrative shadows (arrowheads) can be seen in the left lower lung field

**FIGURE 2:**
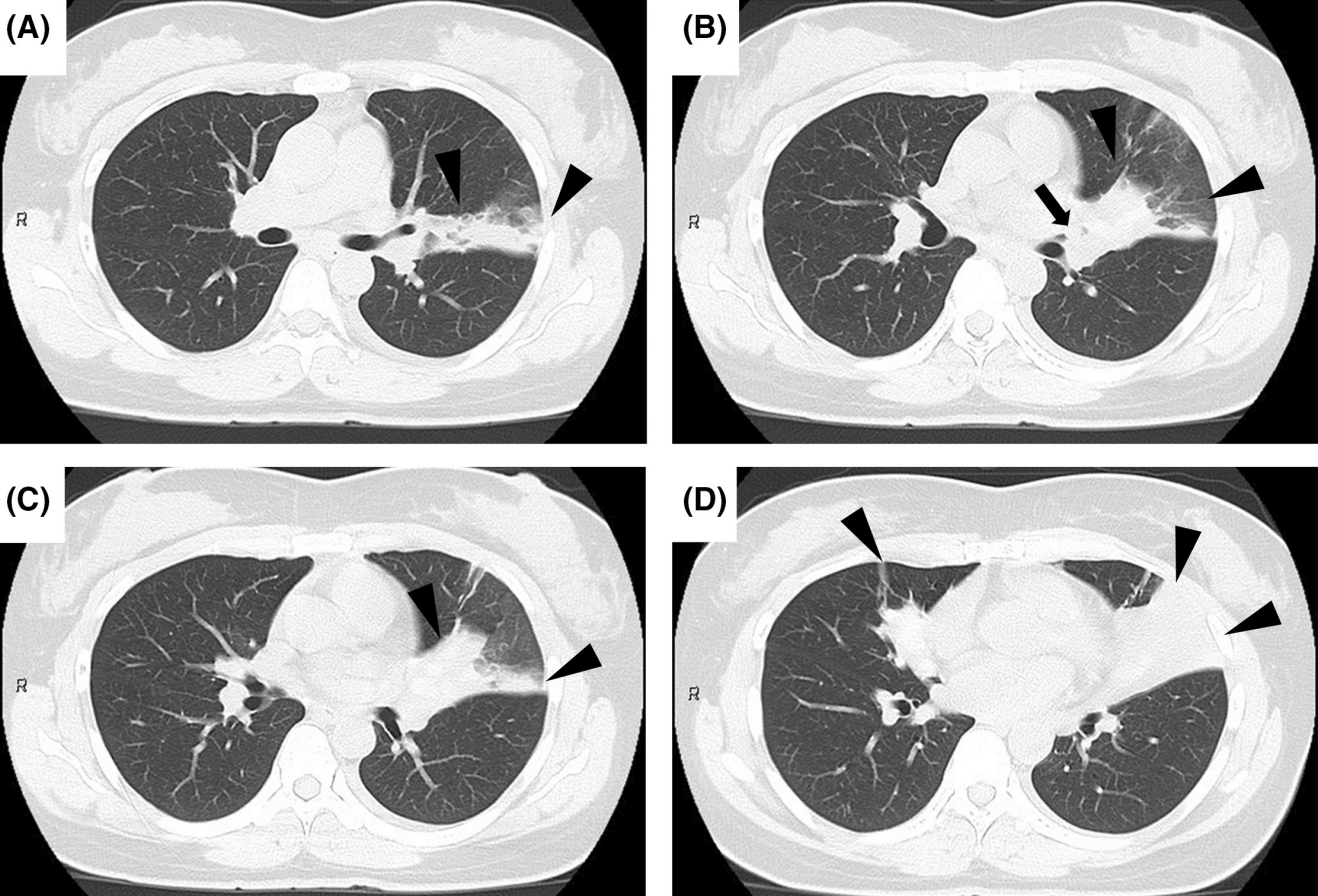
Simple chest computed tomography scan obtained on admission (lung window). Infiltrative shadows are present in the left upper lobe and right middle lobe ((A–D) arrowheads). A mucus plug can be seen in the left upper lobe bronchus ((B) arrow)

**FIGURE 3:**
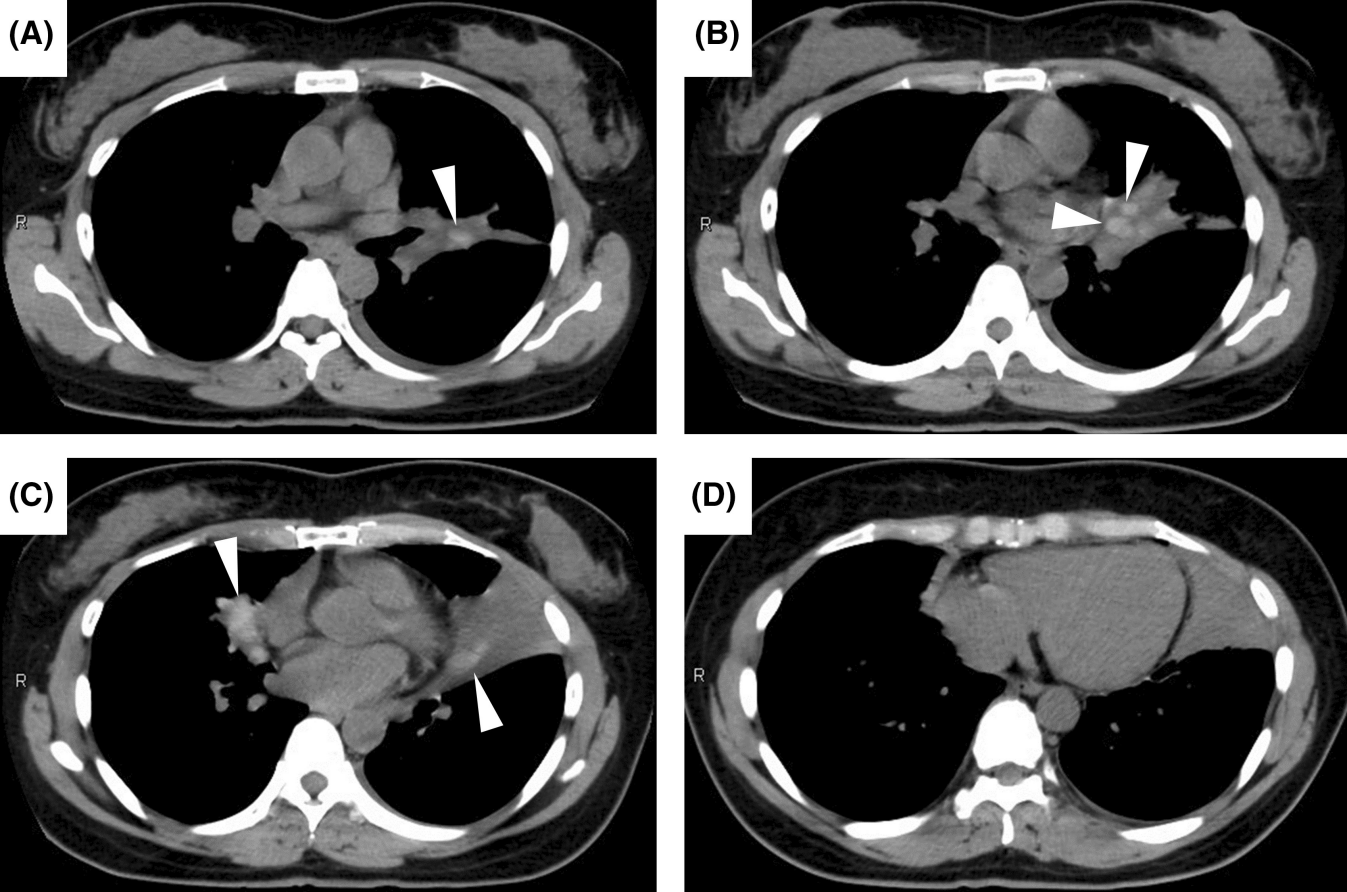
Simple chest computed tomography scans obtained on admission (mediastinal window). High‐attenuation mucus is visible in the left upper lobe bronchus and the right middle lobe bronchus ((A–C) arrowheads)

A 45‐year‐old woman with a history of asthma visited our hospital with chest pain from the previous day. Physical examination revealed tachypnea, percutaneous oxygen saturation of 98% on room air, and diminished breath sounds in the left lower lung field. A chest radiograph and plain chest computed tomography (CT) revealed infiltrating shadows in the left upper and right middle lobe (Figures 1 and 2, arrowheads). Treatment with intravenous ampicillin‐sulbactam for 1 week was unsuccessful. Laboratory tests subsequently became available and showed the following: eosinophils 1,343 /μL, immunoglobulin E 1,010 IU/mL, and β‐d‐glucan 194 pg/mL. Retrospective review of CT scans obtained on admission revealed high‐attenuation mucus (HAM) in the left upper and right middle lobe bronchi (Figure 2B, arrow; Figure 3A–C, arrowheads). Therefore, the patient met the criteria for a diagnosis of allergic bronchopulmonary mycosis (ABPM).^1^ She was started on prednisolone 40 mg/day and her symptoms and laboratory parameters improved.

Although we did not recognize HAM on CT initially, its presence has a sensitivity of 39.7% and a specificity of 100% for ABPM.^2^ For early diagnosis and treatment of ABPM, it is important to check for HAM on CT and be aware of its high specificity for ABPM.

## AUTHOR CONTRIBUTIONS

RH was involved in the literature search, study conception, and drafting of the manuscript. MT was involved in the literature search, study conception, and drafting and revision of the manuscript. SM was involved in the literature search and study conception. TO was involved in the literature search and clinical care of the patient. YO was involved in the literature search, clinical care of the patient, and revision of the manuscript.

## CONFLICT OF INTEREST

The authors stated that they have no conflict of interest.

### ETHICAL APPROVAL

This manuscript conforms to the provisions of the Declaration of Helsinki in 1995 (as revised in Brazil 2013).

### CONSENT

Written informed consent was obtained from the patient to publish this report in accordance with the journal's patient consent policy.

## Data Availability

The data that support the findings of this study are available from the corresponding author upon reasonable request.
